# Cervical cancer screening intervals extendable to ten years for 9v HPV vaccinated persons

**DOI:** 10.1016/j.lana.2026.101486

**Published:** 2026-04-27

**Authors:** Diane M. Harper, Alisa P. Young

**Affiliations:** University of Michigan, MI, USA

As deBondt et al. highlight,^1^ the *screening intervals* between negative high-risk human papillomavirus (hrHPV) primary testings are one part of cervical cancer screening that remains unsettled. Their study showed that de-intensification of screening intervals is needed for optimal screening of HPV-vaccinated US populations.

The *technique* of screening is firmly established. As of January 2026, primary HPV testing for cervical cancer screening is the preferred method of screening. In the US, the Women's Preventive Services Initiative (WPSI), backed by the US Health Research and Services Administration (HRSA) and the Affordable Care Act (ACA) which legislates payment for cervical cancer screening, specifically states that primary HPV testing is the preferred test and that all women, not just those with no access or underserved, should be offered self-sampling as the first option for screening. Globally, the World Health Organization has promoted primary HPV screening since 2021.^2^ Primary HPV testing begins the molecular biomarker screening era. Cervical cancer screening cost-effectiveness models using detection systems for HPV genotypes and biomarkers for cellular oncogenesis are signaling more cost-effectiveness than using a cytology triage.^3^

The *ages of initiation and cessation* remain in flux. *Initiation* of cervical cancer screening should coincide with the age at which there is a rapid uptick in incidence in cervical intraepithelial neoplasia grade 3 or worse (CIN 3+). On average, persistent HPV infection takes fifteen years to transform to CIN3+, meaning that exposure to HPV would be expected at six years of age for a 21-year screening initiation age, or ten years of age for a 25-year screening initiation, or 15 years of age for a 30-year screening initiation to balance the benefits vs the harms of screening. deBondt's modeling supports 30 years of age for screening initiation among the 9vHPV vaccinated; 27 years of age if the 2v/4vHPV vaccine returned to use; and 25 years for the unvaccinated.

*Cessation* age continues to be modeled at 65 years based on the assumption that an 80-year-old woman has a greater likelihood of dying from other diseases than cervical cancer. However, in the US, 21% of cervical cancers are detected in women older than 65 years,^4^ often because of a lack of prior screening. deBondt's model assumed that all women were screened, that they perfectly adhered to follow-up recommendations, and that vaccination had 95% lifetime efficacy; yet, none of these assumptions is accurate. While this methodological fault creates higher than realistic expectations of CIN3+ prevention, the impact of HPV vaccination may improve the prevention of disease by 2070, when all women then 65 and younger would have been offered the opportunity for HPV vaccination, whose efficacy continues beyond 15 years.^5^

The screening interval between negative hrHPV tests among vaccinated women is the highlight of this model. The screening interval between negative hrHPV tests in the vaccinated cohorts (2v/4v or 9v) supported lengthening the intervals between screening compared to current practice, suggesting 8–10 year intervals. This lengthened screening interval maintains the cost-effectiveness of balancing the harms and benefits of screening with disease detection. This is practice-changing.

At the same time, we know that the other seven non-vaccine high-risk HPV types continue to infect women and men, replacing the vaccine-related HPV types within eight years of vaccination,^6^ allowing CIN3+ to occur, but at a slower rate due to the less aggressive nature of the other HPV types.^5^^,^^7^ The non-HPV vaccine type infections progressing to cancer still fit within the current modeling study extending the negative HPV screening interval, which includes all 14 HPV types, to ten years.

In addition, we know that in the US, the rate of cervical cancer among women 18–34 years old has now, for the first time, registered at 3.7/100,000,^8^ below the 4/100,000 incidence that equates to the WHO's cervical cancer elimination goal.^2^ This is due to the 57% HPV vaccination uptake of men and women currently 18–34 years old in the US.^9^ In the US, 30% of the population does not screen for cervical cancer, with even greater unscreened proportions for women 50–65 years.^10^ As vaccination uptake increases and primary HPV self-sampling allows a greater screened US population, the US can reach the WHO goal of reducing cervical cancer to 4/100,000 women using an initiation age of 25 and a safe interval between cervical cancer screenings of every 10 years.

## Contributors

All authors' contributions were equal in conception, writing, editing, and approval. All authors have read and approved the final version of the manuscript.

## Declaration of interests

The authors declared no conflicts of interest.
